# Supplementary material for: Subtle Changes at the RBD/hACE2 Interface During SARS-CoV-2 Variant Evolution: A Molecular Dynamics Study
Source: Biomolecules. 2025 Apr 7;15(4):541. doi: 10.3390/biom15040541 (PMC12024731; doi:10.3390/biom15040541)
Supplement: Supplementary file 1 [file biomolecules-15-00541-s001.zip › biomolecules-3515097-supplementary.pdf]

Supporting Information

Subtle changes at the RBD/hACE2 interface during SARS-CoV-2 variant evolution: a molecular dynamics study

Aria Gheeraert [1,2#], Vincent Leroux [3#], Dominique Mias-Lucquin [3], Yasaman Karami [3], Laurent Vuillon [1], Isaure Chauvot de Beauchêne [3], Marie-Dominique Devignes [3], Ivan Rivalta [2,4], Bernard Maigret [3,*] and Laurent Chaloin [5,*]

Supp. Table S1: Current SARS-CoV-2 Omicron strains and occurring mutations in RBD

Supp. Table S2: Evolution of mutations between past important SARS-CoV-2 strains prior to Omicron

Supp. Table S3: Evolution of mutations in spike RBD for of all major SARS-CoV-2 strains

Supp. Table S4: Covariance analysis on 3D coordinates for each system

Supp. Table S5: Non-exhaustive list of computational and experimental studies reporting binding affinity measurements or calculations between hACE2 and spike RBD

Supp. Fig. S1: Amino acid sequences and structural secondary elements of spike RBD variants

Supp. Fig. S2: Comparison of 2D-RMSD maps of RBD WT in the presence or in the absence of glycan.

Supp. Fig. S3: Complete interactions profiles computed by the PairInt procedure for all strains.

Supp. Fig. S4: Simplified profiles of non-bonded interactions calculated by the PairInt procedure for three additional replicas of WT in the absence (A) or in the presence of glycans (B).

Supp. Fig. S5: Detailed view of all hydrophobic interactions detected during the entire simulation

Supp. Fig. S6: cPCA scree plot showing the evolution of the explained variance ratio for each PC

Supp. Fig. S7: Time-evolution of the PC1-6 values taken during each simulation for each system

Supp. Fig. S8: Average nLMI values for all the studied systems

Supp. References

Supp. Table S1: Current SARS-CoV-2 Omicron strains and occurring mutations in RBD region from residues 333 to 526 (as of 2024-09-23)

Compared to the original Hu-1/19A SARS-CoV-2 strain, all major Omicron strains share the same 8 mutations on spike RBD that are not represented in this table: 4 of those previously observed: **K417N** (Beta/20H, AY.1-3 Delta single mutants), **N440K** (B.1.619), **S477N** (20F, B.1.160, B.1.526.2, B.1.620) and **N501Y** (Alpha/20I, Beta, Gamma/20J, Theta/21E, Mu/21H); 4 additional mutations can be considered Omicron-specific: **S373P**, **S375F**, **Q498R**, and **Y505H**. Note: since XBB and sub-variants eventually retrieved Lambda/21G-specific mutation F490S, all defining mutations on major strains pre-Omicron have been found again in at least one Omicron major strain. Most major Omicron strains further share 6 mutations not shown here: **S371F**, **T376A**, **D405N**, **R408S** (new), **E484A** (frequently mutated pre-Omicron but not into alanine), and **T478K** (Delta/21A-defining).

**Code**	**Pango (Nextstrain) designation, initial detection**	**Strain-distinguishing SPIKE RBD (333-526) mutations in the Omicron family**													
**Hu-1**	original SARS-CoV-2 strain **B (19A)** – Wuhan, China, 2019-12	**G** 339	**R** 346	**L** 368	**K** 444	**V** 445	**G** 446	**L** 452	**L** 455	**F** 456	**N** 460	**F** 486	**F** 490	**Q** 493	others
**BA.1**	**Omicron (21M) sub-clade BA.1 (21K)** – Botswana, 2021-11	D					S							R	S371L T376 D405 R408 G496S
**BA.2**	**Omicron BA.2 (21L)** – South Africa, 2021-11	D												R	
**BA.2.12.1**	**Omicron BA.2.12.1 (22C)** – USA, 2021-12	D						Q						R	
**BA.4/5**	**Omicron BA.4 / BA.5 (22A / 22B)** – South Africa, 2022-01/02	D						R				V			
**BA.2.75**	**Omicron BA.2.75 (22D)** – India, 2022-05 – "Centaurus"	H					S				K				
**BA.2.75.2**	**Omicron BA.2.75.2** – India, 2022-07	H	T				S				K	S			
**BQ.1.1**	**Omicron BQ.1 (22E)** (BA.5.3.1.1.1.1.1.1) – Nigeria, 2022-08	D	T		T			R			K	V			
**XBB**	**Omicron XBB / XBB.1.9 (22F / 23D)** – India, 2022-08	H	T	I		P	S				K	S	S		
**XBB.1.5**	**Omicron XBB.1.5 / XBB.2.3 (23A / 23E)** – USA, 2022-10	H	T	I		P	S				K	P	S		
**XBB.1.16**	**Omicron XBB.1.16 (23B)** – Asia, 2023-01 – "Arcturus"	H	T	I		P	S				K	P	S		T478R
**CH.1.1**	**Omicron CH.1.1 (23C)** – 2022-09	H	T		T		S	R			K	S			
**EG.5**	**Omicron EG.5.1 (23F)** – Israel, 2023-02 – "Eris"	H	T	I		P	S			L	K	P	S		
**DV.7.1**	**Omicron DV.7.1** – Austria/Spain, 2023-05	H	T		T		S	R	F	L	K	S			
**HK.3**	**Omicron HK.3** – also GK.* – China, 2023-06	H	T	I		P	S		F	L	K	P	S		
**BA.2.86**	**Omicron BA.2.86 (23I)** – Denmark/Israel, 2023-07 – "Pirola"	H	T			H	S	W			K	P		R	R403K N450D N481K 483del **E484K**
**JN.1/1.11.1**	**Omicron JN.1 (24A)** – Europe/India, late 2023, early 2024	H	T			H	S	W			K	P		R	
**KP.3**	**Omicron KP.3 (24C)** – India, early 2024	H	T			H	S	W			K	P		E	

Supp. Table S2: Evolution of mutations between past important SARS-CoV-2 strains prior to Omicron arrival (ECDC as of 2024-09-23).

Color coding: yellow cases indicate specific mutations among the major strains presented here; orange are mutations of particular interest in discussing of recent (2022) Omicron RBD mutations; green and blue correspond to mutations shared in the whole Omicron family, the former not being observed previously (E484 was mutated frequently but not into A) contrary to the latter. For Omicron and since 2024, more mutations were described and are not represented here (see Table S3 for more details).

Code	Major de-escalated variants (ECDC: 2023-03-03)	Most-observed pre-Omicron SPIKE RBD mutations (333-526)								
		R346	K417	L452	S477	T478	E484	F490	N501	Y505
Α	**Alpha (20I)** B.1.1.7 – UK 2020-09								Y	
Β	**Beta (20H)** B.1.351 – South Africa 2020-05		N				K		Y	
Γ	**Gamma (20J)** P.1 – Brazil 2020-11<br>P.1+P681H – Italy 2021-02		T				K		Y	
Ε	**Epsilon (21C)** B.1.427/429 – California 2020-09<br>Also: C.16 – 2020-10 ; C.36+L452R – Egypt 2020-12 ; B.1.526.1 – USA 2020-10			R						
Ζ Η Ι	**Zeta** P.2 – Brazil 2021-01 ; R.1 – Japan 2020-10 ;<br>**Eta (21D) / Iota (21F)**, B.1.525/526 – Nigeria / USA, 2020-12<br>Also: B.1.1.318 – 2021-01 ; B.1.1.519 (20B/S.732A) – 2020-11 ; AT.1 – Russia 2021-01						K			
Θ	**Theta (21E)** P.3 – Philippines 2021-01<br>B.1.1.7+E484K – UK 2020-12						K		Y	
Κ	**Kappa (21B)** B.1.617.1 – India 2020-12<br>B.1.617.3 – India 2021-02			R			Q			
Δ	**Delta B.1.617.2 (21A, 21I, 21J)** – India 2020-10, VOC until mid-2022<br>AY.4.2 (B.1.617.2.4.2) – UK 2021-06<br>B.1.617.2+Q613H and B.1.617.2+Q677H – India 2021-04			R		K				
Λ	**Lambda (21G)** C.37 – Peru 2020-12			Q				S		
Μ	**Mu (21H)** B.1.621 – Colombia 2021-01	K					K		Y	
Ο	*For reference:* **Omicron** – dominant VOC in 2023	K<br>BA.1.1	N	R<br>BA.4/5	N	K	A/K		Y	H

Supp. Table S3: Evolution of mutations in spike RBD for of all major SARS-CoV-2 strains known as of 2024-09-23. The Spike RBD is defined as residues in the 333-526 sequence. Each strain is designated by its common name (WHO), its Pango designation and its Nextstrain code (when available). Omicron BA.4/5 share the same RBD sequence. All structural models (PDB) correspond to complex RBD/hACE2 (1:1). Mutations are color-coded as follows: yellow mutations are specific among the major ones presented here; green are Omicron-specific and shared by all Omicron sub-variants; blue are also shared by all Omicron sub-variants but were previously identified; orange are mutations that are of particular interest upon discussion of recent Omicron RBD mutations.

Strain designation			Models	Mutations on SPIKE RBD sequence (333-526)																									
common name	Pango	Nextstrain	PDB	339	346	371	373	375	376	405	408	417	440	444	446	450	452	460	477	478	484	486	490	493	494	496	498	501	505
**Hu-1**	B	**19A**	6M0J	**G**	**R**	**S**	**S**	**S**	**T**	**D**	**R**	**K**	**N**	**K**	**G**	**N**	**L**	**N**	**S**	**T**	**E**	**F**	**F**	**Q**	**S**	**G**	**Q**	**N**	**Y**
Alpha	B.1.1.7	20I	7EKF																									Y	
Beta	B.1.351	20H	7EKG									N									K							Y	
Gamma	P.1	20J	7EKC									T									K							Y	
Epsilon	B.1.427	21C	7SY0														R												
Eta / Iota / Zeta	B.1.525, P.2	21D 21F																			K								
Theta	P.3	21E	7SY4																		K							Y	
Kappa	B.1.617.1	21B	7TEZ														R				Q								
**Delta**	**B.1.617.2**	**21A**	7WBQ														R			K									
Lambda	C.37	21G															Q						S						
Mu	B.1.621	21H			K																K							Y	
**Omicron BA.1**	B.1.1.529.1	**21K**	7T9L	D		L	P	F				N	K		S				N	K	A			R		S	R	Y	H
Omicron BA.1.1			7XAZ	D	K	L	P	F				N	K		S				N	K	A			R		S	R	Y	H
**Omicron BA.2**		**21L**	7XB0	D		F	P	F	A	N	S	N	K						N	K	A			R			R	Y	H
Omicron BA.2.3.20				D		F	P	F	A	N	S	N	K	R		D	M	K	N	K	R						R	Y	H
Omicron BA.2.10.4				D		F	P	F	A	N	S	N	K		S				N	K	A	P			P		R	Y	H
Omicron BA.2.12.1		22C	7HYW	D		F	P	F	A	N	S	N	K				Q		N	K	A			R			R	Y	H
Omicron BA.2.75		22D	8H5C	H		F	P	F	A	N	S	N	K		S			K	N	K	A						R	Y	H
Omicron BA.2.75.2				H	T	F	P	F	A	N	S	N	K		S			K	N	K	A	S					R	Y	H
Omicron BA.3			7XB1	D		F	P	F		N		N	K		S				N	K	A			R			R	Y	H
**Omicron BA.4 / BA.5**		**22A 22B**	7XWA	D		F	P	F	A	N	S	N	K				R		N	K	A	V					R	Y	H
Omicron BA.4.6				D	T	F	P	F	A	N	S	N	K				R		N	K	A	V					R	Y	H
Omicron BQ.1.1		22E	8FXC	D	T	F	P	F	A	N	S	N	K	T			R	K	N	K	A	V					R	Y	H
Omicron XAK				D	K	F	P	F	A	N	S	N	K					K	N	K	A						R	Y	H
Omicron BA.2.86		23I		D	K	F	P	F	A	N	S	N	K					K	N	K	K						R	Y	H
Omicron JN.1 (1.11.1)		24A /24B		D	K	F	P	F	A	N	S	N	K					K	N	K	K						R	Y	H
Omicron KP.3		24C		D	K	F	P	F	A	N	S	N	K					K	N	K	K			E			R	Y	H

Supp. Table S4: Covariance analysis on 3D coordinates for each system to evaluate the contribution of the first PCs.

**PC n°**	**WT**	**Delta**	**BA.1**	**BA.1^{+Q493K}**	**BA.2**	**BA.4**
**1**	0.40	0.28	0.36	0.26	0.37	0.39
**2**	0.15	0.19	0.09	0.16	0.19	0.18
**3**	0.09	0.11	0.07	0.12	0.08	0.07
**4**	0.06	0.07	0.06	0.08	0.05	0.05
**5**	0.03	0.04	0.05	0.05	0.03	0.03
**6**	0.02	0.03	0.03	0.05	0.02	0.02
**7**	0.02	0.03	0.03	0.04	0.02	0.02
**8**	0.01	0.02	0.02	0.04	0.02	0.01
**9**	0.01	0.01	0.02	0.02	0.01	0.01
**10**	0.01	0.01	0.02	0.02	0.01	0.01
Cumulative var. (1-10)	0.80	0.79	0.75	0.84	0.8	0.79
**Cumulative var. (1-2)**	**0.55**	**0.47**	**0.45**	**0.42**	**0.56**	**0.57**

Supp. Table S5: Non-exhaustive list of computational and experimental studies reporting RBD binding affinity to hACE2 for ancestral strain (WT) or variants (ND: Not Done; NA: Not applicable).

**SARS-CoV-2**	**Method ***	**Simulation time**	**RBD binding affinity to hACE2 (experimental or computed)**		**Ref.**
			**Variant vs RBD^{WT}**	**Variant vs RBD^{δ}**	
wt, o	MD, MM/GBSA	2x500 ns	Stronger affinity of RBD^{O} than RBD^{WT}	ND	[1]
wt, o	MD	50 ns	More stable binding for RBD^{O}. Partial dissociation with RBD^{WT}	ND	[2]
wt, o	MD, FEP	20 ns	Stronger affinity of RBD^{O} than RBD^{WT} (factor of 2.5)	ND	[3]
wt, o	Docking, MM/GBSA	400 - 500 ns	Stronger affinity of RBD^{O} than RBD^{WT} (factor of 2)	ND	[4]
wt, o	MD, MM/PBSA	3x100 ns	Stronger affinity of RBD^{O} than RBD^{WT} (factor of 2.5)	ND	[5]
wt, o	MD, MM/PBSA	100 ns	Equivalent affinity of RBD^{O} and RBD^{WT}	ND	[6]
wt, δ, o	MD, MM/GBSA / Elisa	200 ns	Stronger affinity of RBD^{O} than RBD^{WT}	Weaker affinity of RBD^{O} than RBD^{δ}	[7]
wt, α, κ, o	MD – Umbrella sampling	500 ns	Stronger affinity of $RBD^{\alpha, \kappa, O}$ than RBD^{WT}		[8]
wt, β, δ, o	SPR	NA	Stronger affinity of RBD^{O} than RBD^{WT} K_D = 13.2 nM (wt), 8.85 nM (omicron)	Weaker affinity of RBD^{O} than RBD^{δ} K_D = 2.85 nM (δ), 8.85 nM (omicron)	[9]
α, β, γ, δ, ε, κ	SMD	20x40 ns	Reinforced interface for RBD^{O} versus RBD^{WT}	ND	[10]
wt, o	MST		Stronger affinity of RBD^{O} than RBD^{WT} (5 and 21.5 nM, respectively)	ND	[11]
wt, α, β, γ, δ, o	X-ray / Cryo-EM / SPR	NA	Similar affinity of RBD^{O} and RBD^{WT} K_D = 25 nM (wt), 31 nM (omicron).	Similar affinity of RBD^{O} and RBD^{δ} K_D = 25 nM (δ), 31 nM (omicron).	[12]
wt, δ, o	BLI	NA	Stronger affinity of RBD^{O} than RBD^{WT} K_D = 127 nM (wt), 44 nM (omicron)	Stronger affinity of RBD^{O} than RBD^{δ}. K_D = 190 nM (δ), 44 nM (omicron)	[13]
wt, α, β, δ, o	SPR	NA	Stronger affinity of RBD^{O} than RBD^{WT} K_D = 60 ± 1.4 nM (wt), 25.3 ± 1.2 nM (omicron)	Stronger affinity for RBD^{O} than RBD^{δ}. K_D = 75 ± 1.9 nM (δ), 25.3 ± 1.2 nM (omicron)	[14]
wt, δ, o	SPR	NA	Stronger affinity (1.4 fold) of RBD^{O} than RBD^{WT}	Similar affinity of RBD^{O} and RBD^{δ}.	[15]

* Abbreviations for computational and experimental methods. AFM: Atomic force microscopy; BLI: Biolayer Interferometry; Cryo-EM: cryo-electron microscopy; FEP: Free-energy perturbation; MD: Molecular dynamics simulations; MM/GBSA: Molecular Mechanics/ Generalized-Born Surface Area; PBSA: Poisson-Boltzmann Surface Area continuum solvation; MST: Microscale thermophoresis; SMD: Steered molecular dynamics; SPR: Surface plasmon resonance.

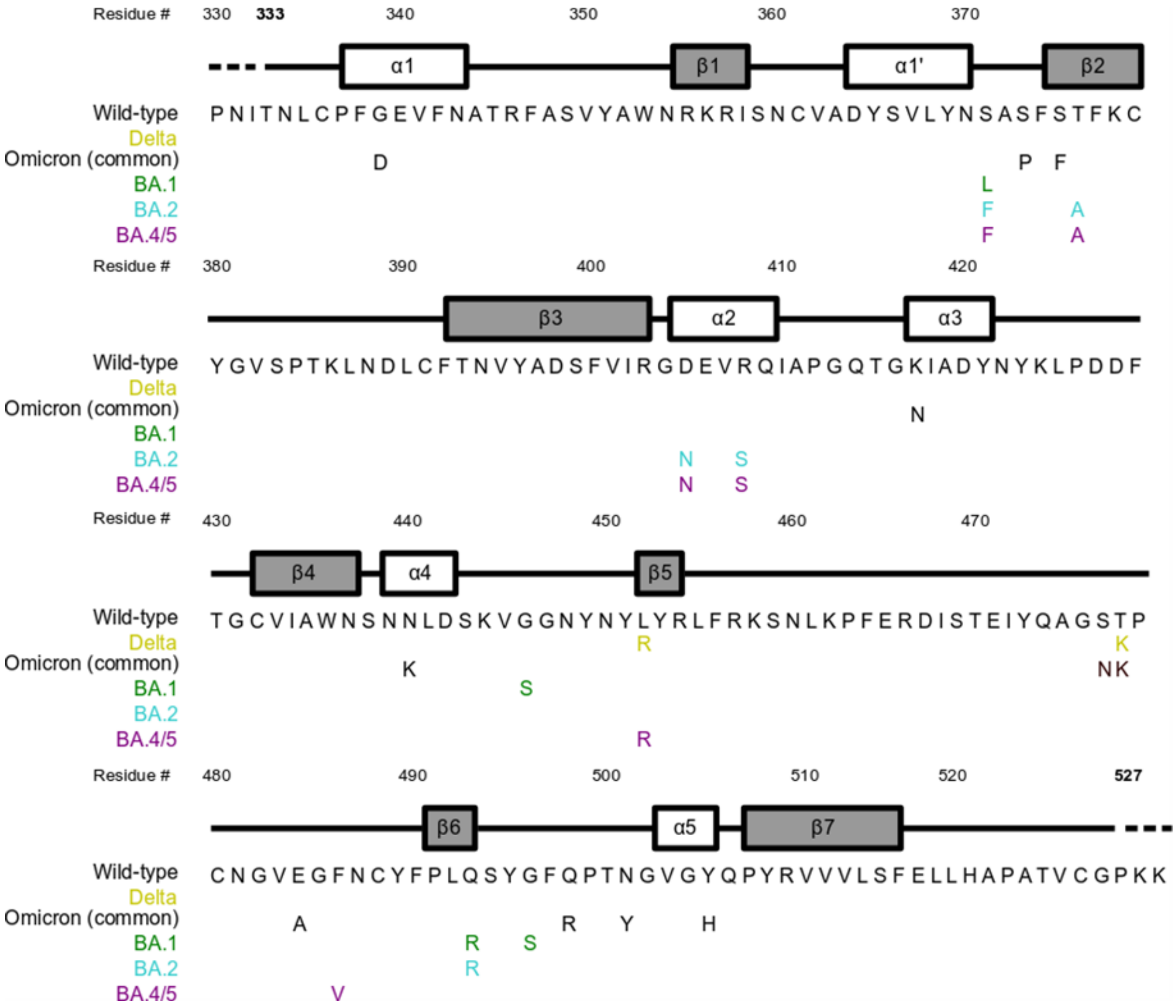

Supp. Fig. S1: Amino acid sequences and structural secondary elements (derived from crystal structure 6M0J[16]) of Spike RBD WT and variants. Mutations found in Delta and Omicron variants (BA.1, BA.2 and BA.4/5) are highlighted in dark yellow, green, cyan and magenta, respectively (BA.1^{+Q493K} is not shown because of its unique difference with BA.1). The common mutations for the Omicron family are related to group 21M (Pango lineage B.1.1.529).

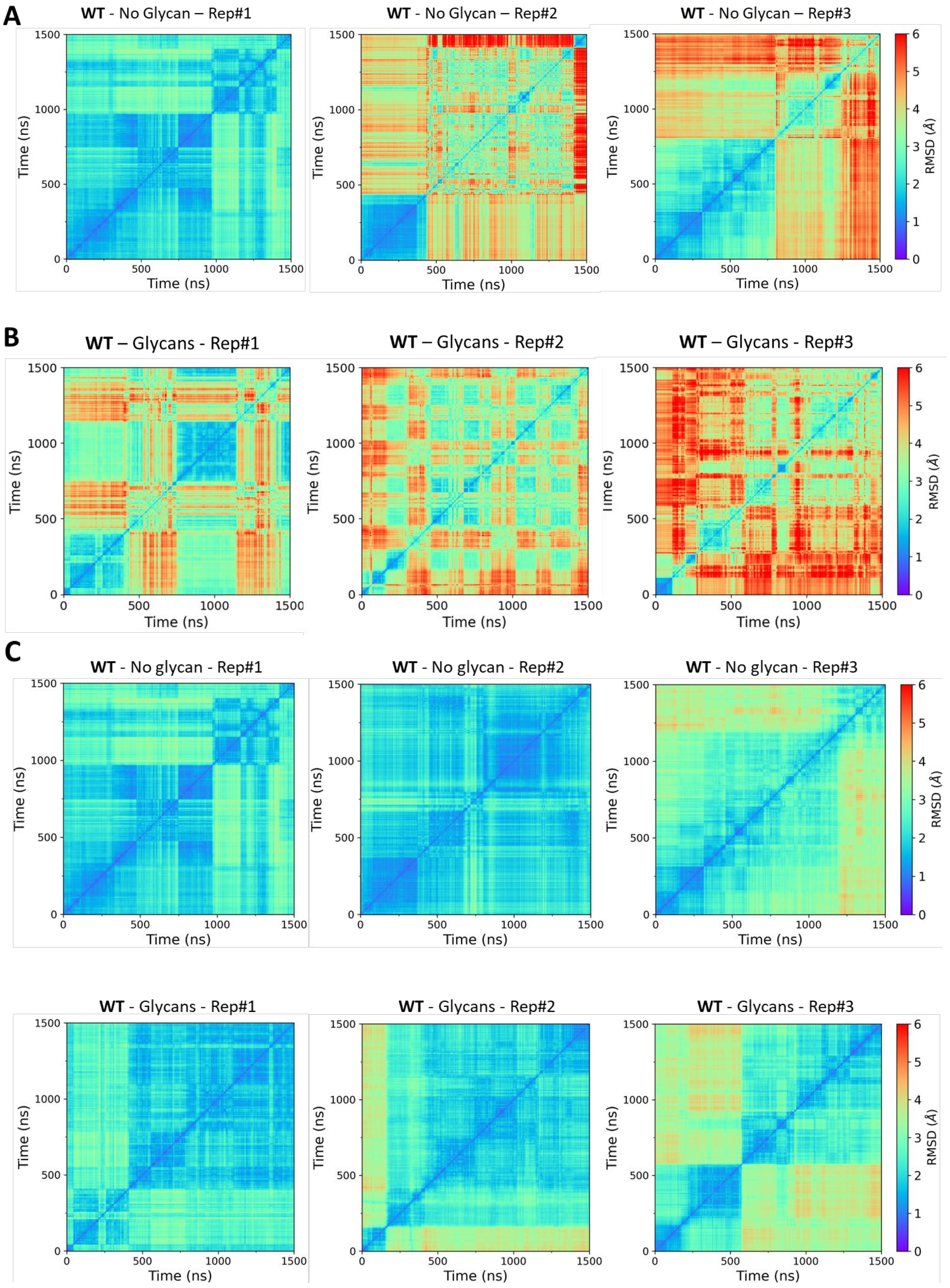

Supp. Fig. S2: 2D-RMSD matrix plots computed for all backbone atoms of RBD WT in the absence (**A**) of in the presence (**B**) of glycans. The increase in RMSD values observed in several replicas was due to a large motion of the c-terminus of RBD (residues 520-526). When computing the same matrix without this c-terminal end, RMSD values did not exceed 3 Å as shown in panel (**C**). Note that in Figure 2 the maximum deviation was 3 Å, whereas here it is defined as 6 Å.

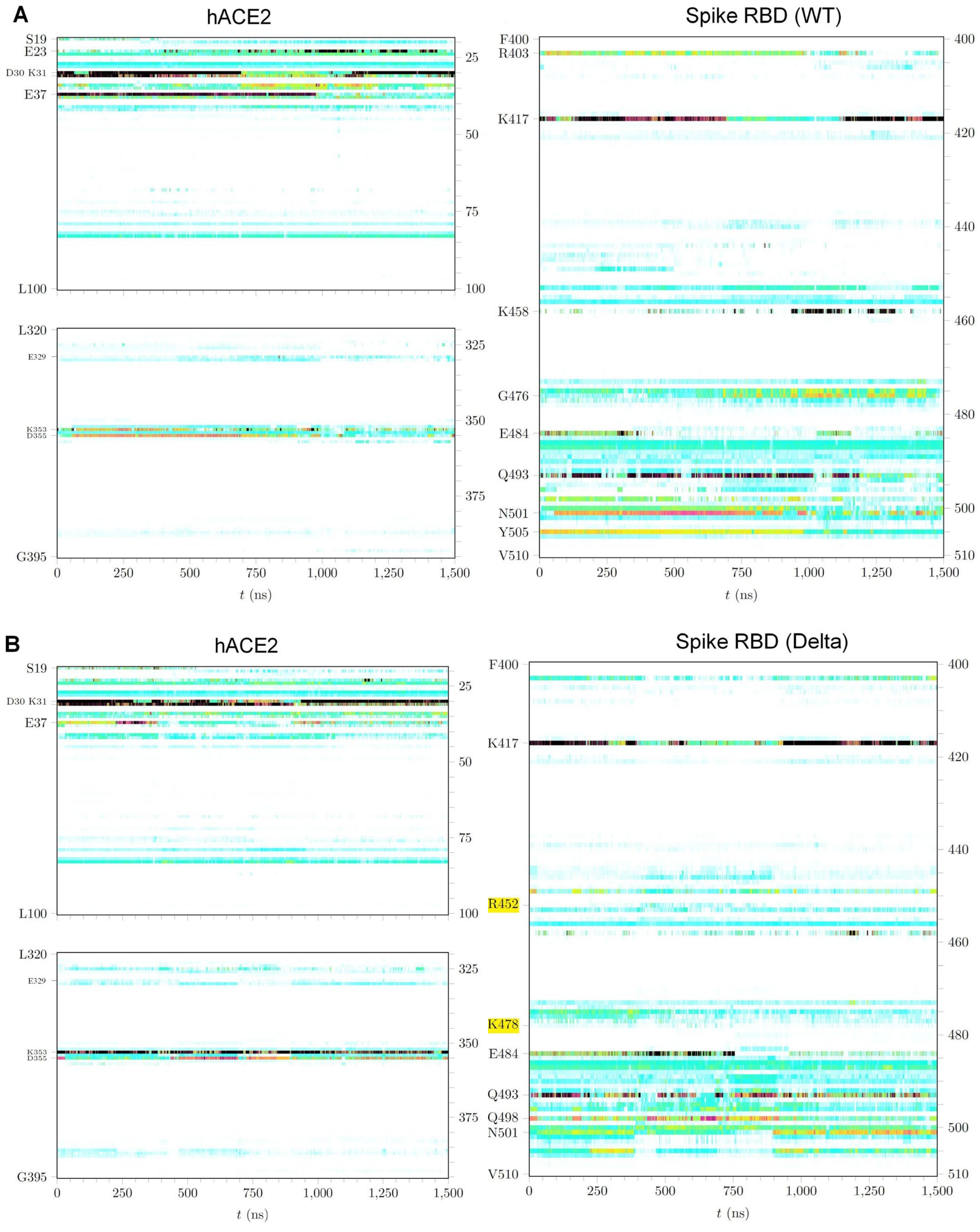
A
hACE2
Spike RBD (WT)
S19
E23
D30 K31
E37
L100
L320
E329
K353
D355
G395
F400
R403
K417
K458
G476
E484
Q493
N501
Y505
V510
t (ns)
B
hACE2
Spike RBD (Delta)
R452
K478
Q498

C

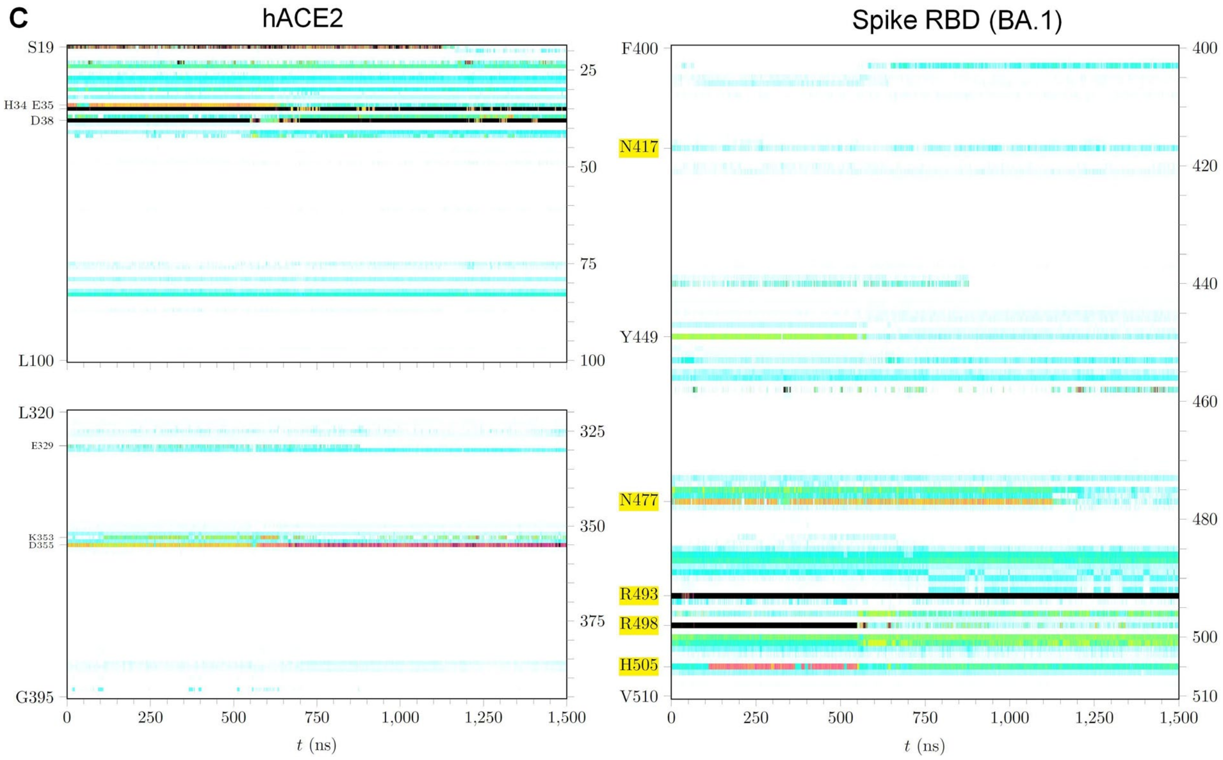

hACE2
S19
H34 E35
D38
L100
L320
E329
K353
D355
G395
25
50
75
100
325
350
375
0
250
500
750
1,000
1,250
1,500
t (ns)
Spike RBD (BA.1)
F400
N417
Y449
N477
R493
R498
H505
V510
400
420
440
460
480
500
510
t (ns)

D

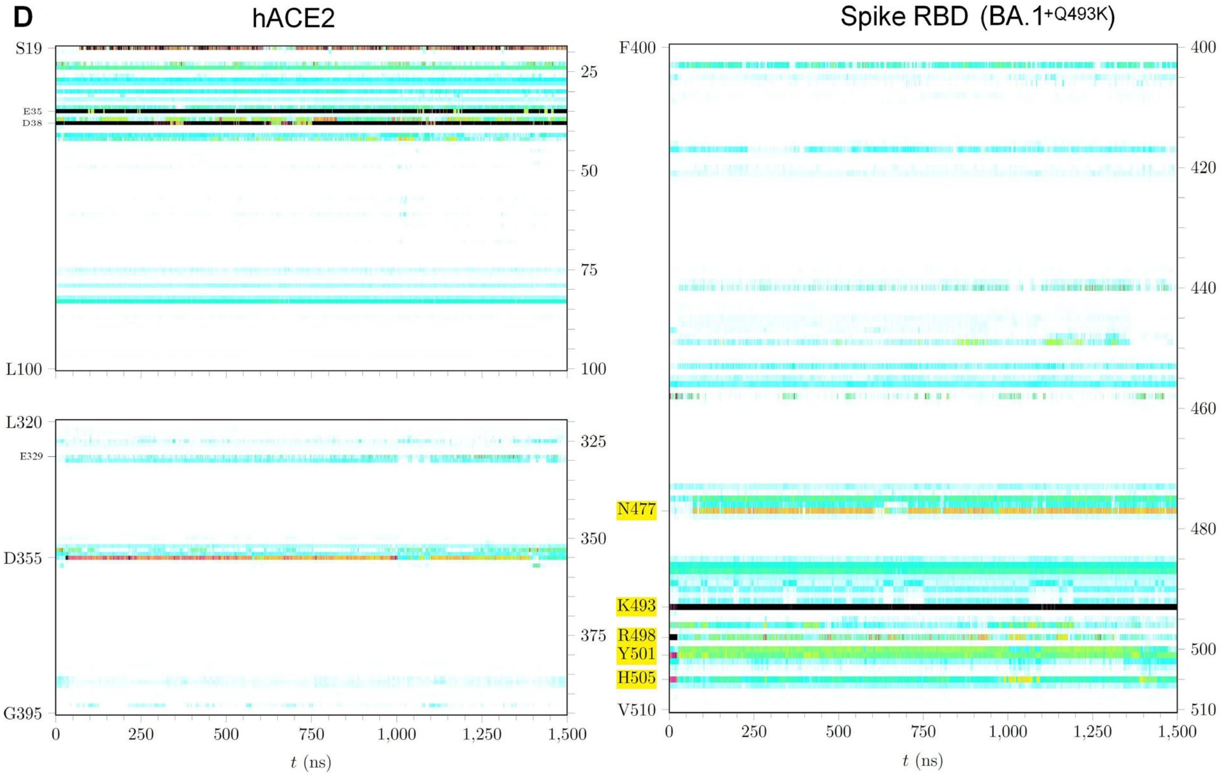

hACE2
S19
E35
D38
L100
L320
E329
D355
G395
25
50
75
100
325
350
375
0
250
500
750
1,000
1,250
1,500
t (ns)
Spike RBD (BA.1+Q493K)
F400
N477
K493
R498
Y501
H505
V510
400
420
440
460
480
500
510
t (ns)

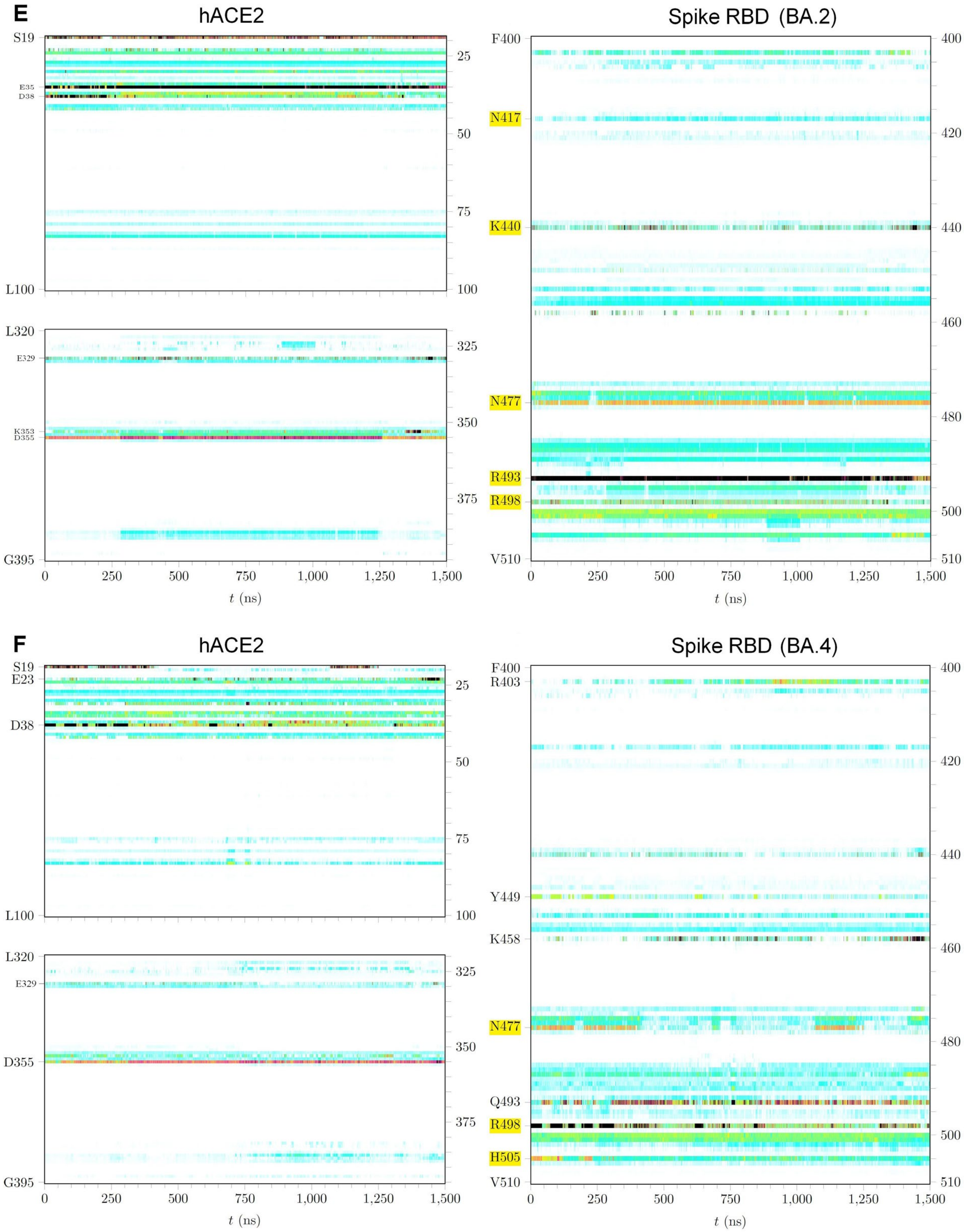

Supp. Fig. S3: Time course of non-bonded interactions calculated by the PairInt procedure showing the implication of both hACE2 and Spike residues for WT (**A**), Delta (**B**), BA.1 (**C**), BA.1^{+Q493K} (**D**), BA.2 (**E**)

and BA.4 (**F**) variants (only sections encompassing residues 400 to 529 are shown for Spike). All interactions were processed by NAMD and homemade scripts using a cutoff distance of 6 Å for electrostatic potential on trajectories unstripped from solvent (same color code for interaction energies as in Fig.4).

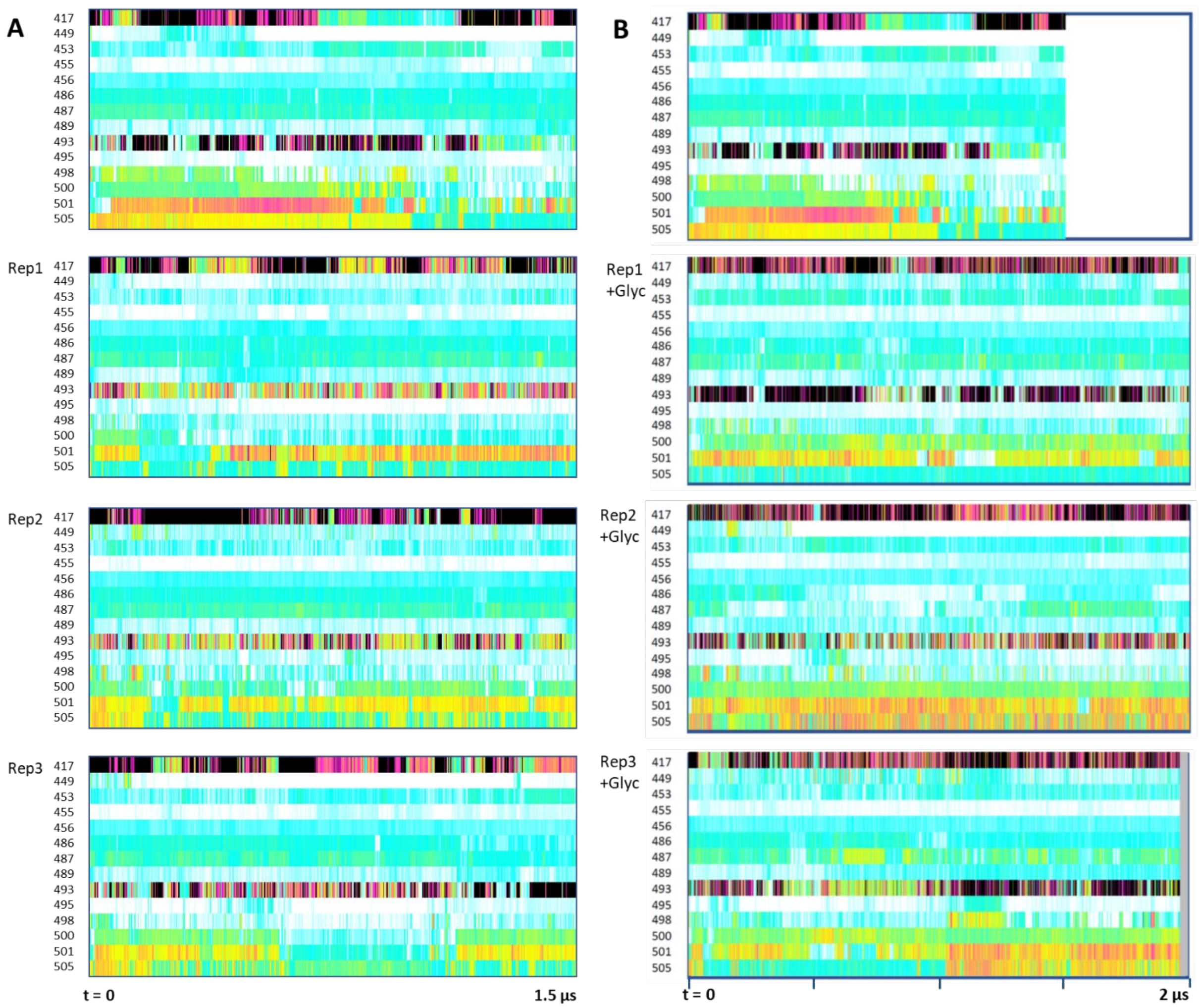

Supp. Fig. S4: Simplified interaction profiles obtained by the PairInt procedure for the WT RBD-hACE2 complex in triplicates on top of the original simulation in the absence (**A**) or in the presence (**B**) of decorating glycans. Note that simulations with glycans were longer and reached 2 µs for each replica.

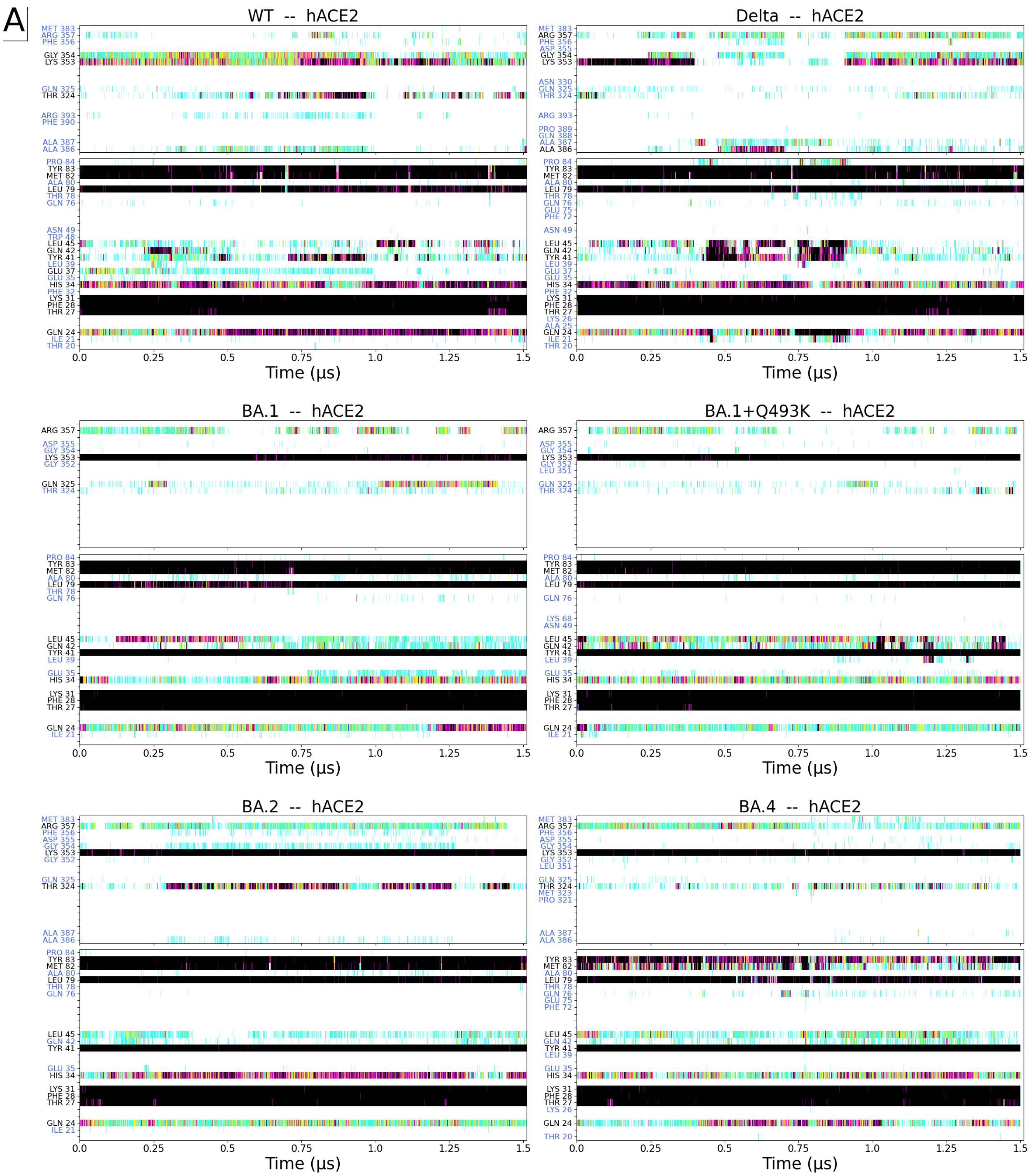

A
WT -- hACE2
Delta -- hACE2
BA.1 -- hACE2
BA.1+Q493K -- hACE2
BA.2 -- hACE2
BA.4 -- hACE2
Time (μs)
0.0
0.25
0.5
0.75
1.0
1.25
1.5

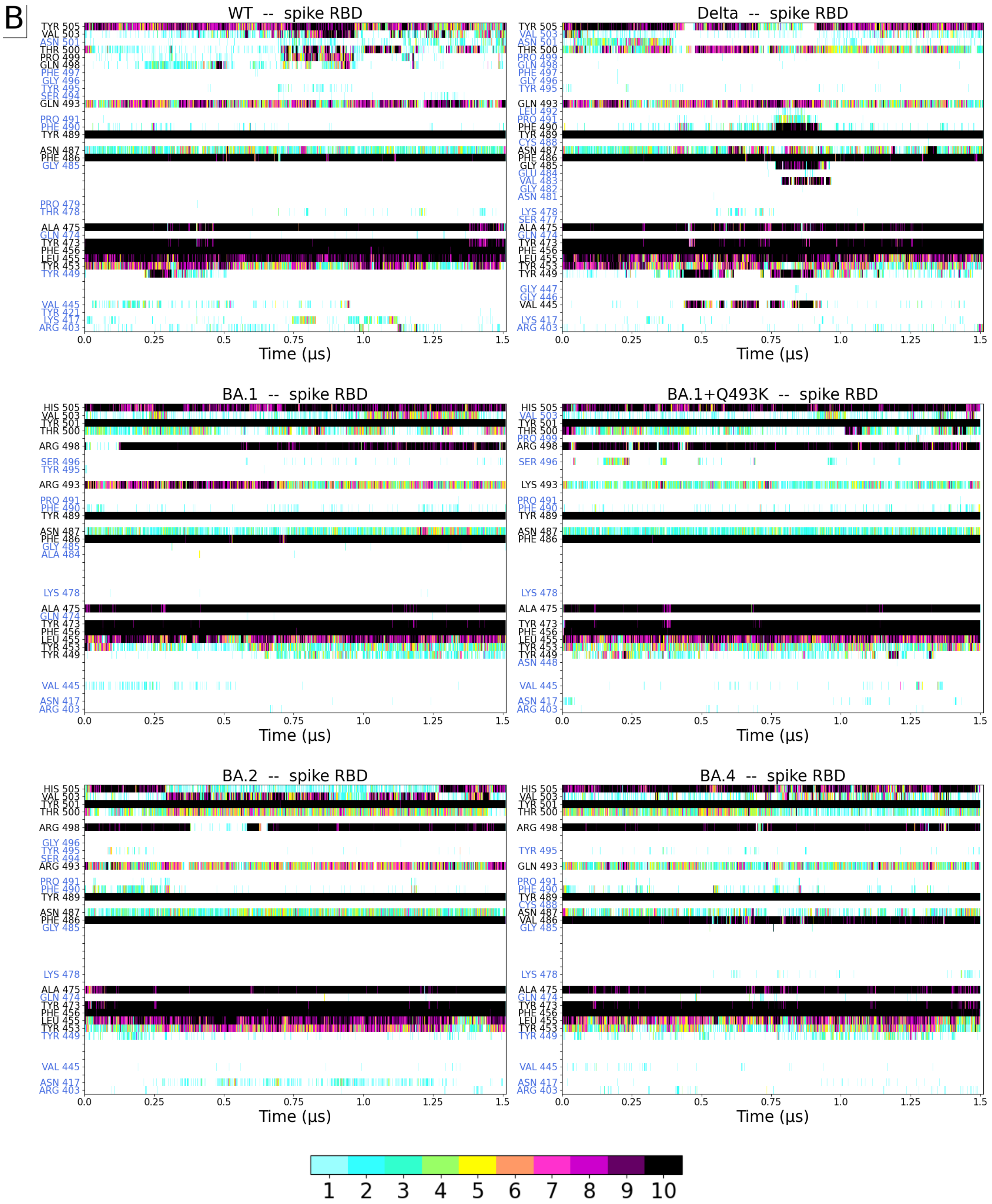

Supp. Fig. S5: Detailed view of all hydrophobic interactions detected during the entire simulation for either (A) Spike residues or (B) hACE2 residues. Each vertical bar on a row represents 10 frames and is depicted in color if the residue is involved in a hydrophobic interaction in 1 (pale blue) to 10 (black) frames. A

residue is only plotted if it is involved in hydrophobic interactions in at least one variant. For each variant, the color of the residue name indicates if it was found interacting in more than 10% of frames (black) or in less than 10% of the frame (blue). Residues not interacting at all in the simulations are not represented for clarity. The scale bar indicates the number of interactions per 10-frame interval.

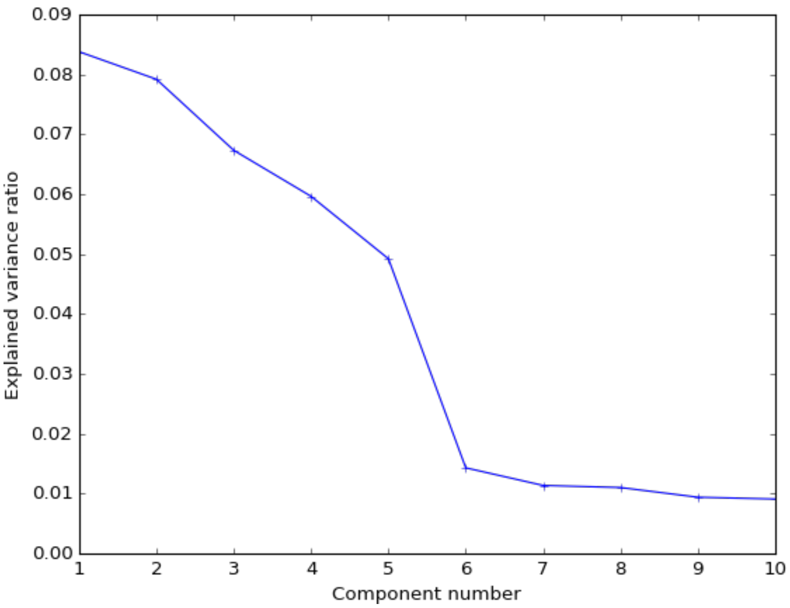

Supp. Fig. S6: cPCA scree plot showing the evolution of the explained variance ratio of each component. Note that in the case of contact-PCA, the values of variances are much lower than those of cartesian-PCA due to the computational procedure.

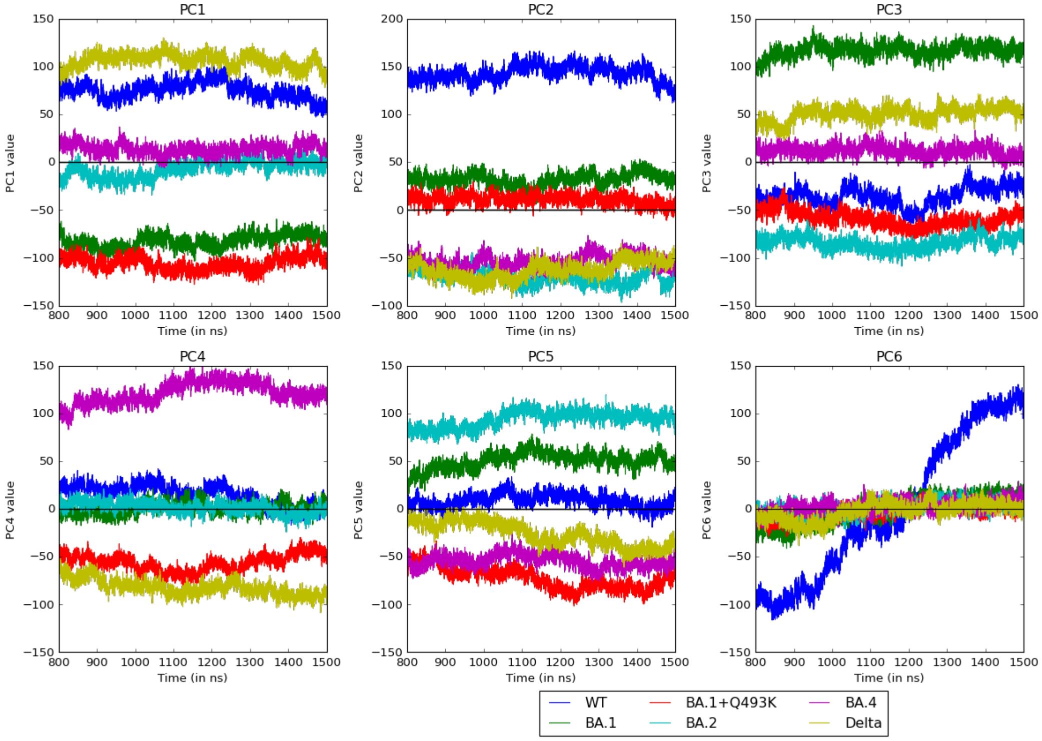

Supp. Fig. S7: Time-evolution of the PC1-6 values taken during each simulation (from 800 to 1500 ns) for each system.

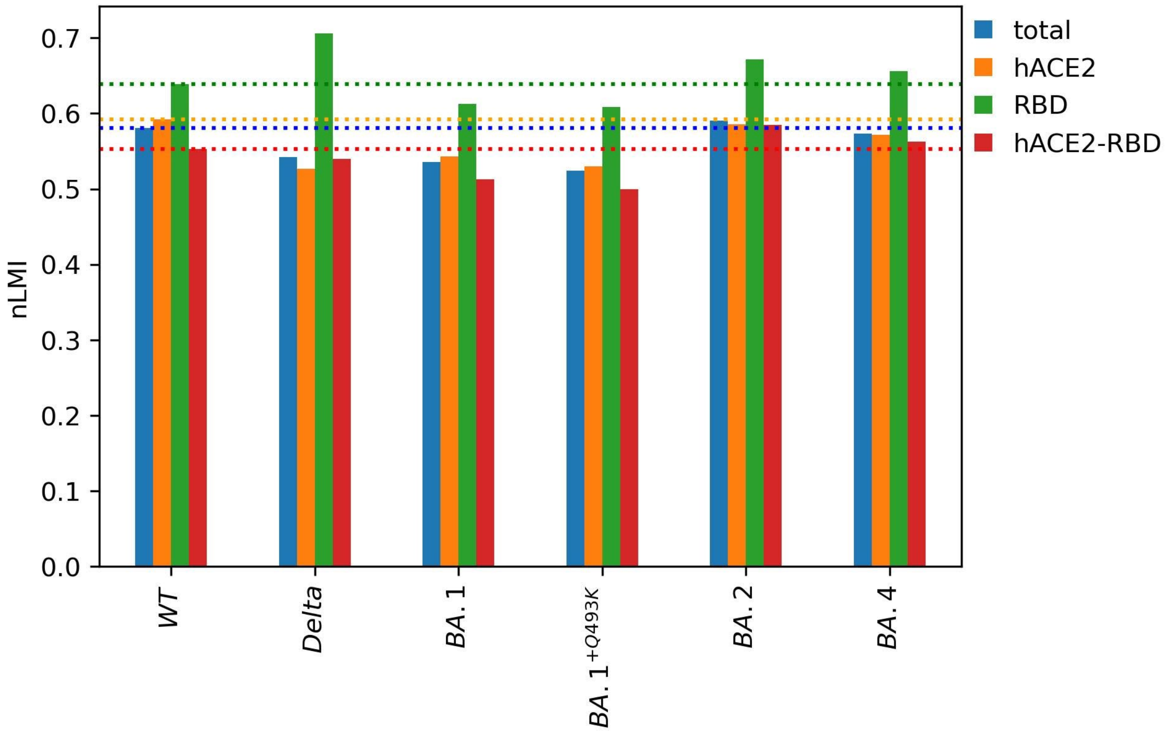

Supp. Fig. S8: Average nLMI values for all the studied systems. The values are averaged over the complete map (in blue), within hACE2 (in orange), within RBD (in green) and between hACE2 and RBD (in red). The horizontal dashed lines depict values of the wild-type system.

References

1. Lupala, C.S.; Ye, Y.; Chen, H.; Su, X.-D.; Liu, H. Mutations on RBD of SARS-CoV-2 Omicron Variant Result in Stronger Binding to Human ACE2 Receptor. *Biochem Biophys Res Commun* **2022**, *590*, 34–41, doi:10.1016/j.bbrc.2021.12.079.
2. Omotuyi, O.; Olubiyi, O.; Nash, O.; Afolabi, E.; Oyinloye, B.; Fatumo, S.; Femi-Oyewo, M.; Bogoro, S. SARS-CoV-2 Omicron Spike Glycoprotein Receptor Binding Domain Exhibits Super-Binder Ability with ACE2 but Not Convalescent Monoclonal Antibody. *Comput Biol Med* **2022**, *142*, 105226, doi:10.1016/j.compbiomed.2022.105226.
3. Shah, M.; Woo, H.G. Omicron: A Heavily Mutated SARS-CoV-2 Variant Exhibits Stronger Binding to ACE2 and Potently Escapes Approved COVID-19 Therapeutic Antibodies. *Front Immunol* **2021**, *12*, 830527, doi:10.3389/fimmu.2021.830527.
4. Khan, A.; Waris, H.; Rafique, M.; Suleman, M.; Mohammad, A.; Ali, S.S.; Khan, T.; Waheed, Y.; Liao, C.; Wei, D.-Q. The Omicron (B.1.1.529) Variant of SARS-CoV-2 Binds to the hACE2 Receptor More Strongly and Escapes the Antibody Response: Insights from Structural and Simulation Data. *Int J Biol Macromol* **2022**, *200*, 438–448, doi:10.1016/j.ijbiomac.2022.01.059.
5. Rath, S.L.; Padhi, A.K.; Mandal, N. Scanning the RBD-ACE2 Molecular Interactions in Omicron Variant. *Biochem Biophys Res Commun* **2022**, *592*, 18–23, doi:10.1016/j.bbrc.2022.01.006.

6. Koley, T.; Kumar, M.; Goswami, A.; Ethayathulla, A.S.; Hariprasad, G. Structural Modeling of Omicron Spike Protein and Its Complex with Human ACE-2 Receptor: Molecular Basis for High Transmissibility of the Virus. *Biochem Biophys Res Commun* **2022**, *592*, 51–53, doi:10.1016/j.bbrc.2021.12.082.
7. Wu, L.; Zhou, L.; Mo, M.; Liu, T.; Wu, C.; Gong, C.; Lu, K.; Gong, L.; Zhu, W.; Xu, Z. SARS-CoV-2 Omicron RBD Shows Weaker Binding Affinity than the Currently Dominant Delta Variant to Human ACE2. *Signal Transduct Target Ther* **2022**, *7*, 8, doi:10.1038/s41392-021-00863-2.
8. Dutta, S.; Panthi, B.; Chandra, A. All-Atom Simulations of Human ACE2-Spike Protein RBD Complexes for SARS-CoV-2 and Some of Its Variants: Nature of Interactions and Free Energy Diagrams for Dissociation of the Protein Complexes. *J Phys Chem B* **2022**, *126*, 5375–5389, doi:10.1021/acs.jpcb.2c00833.
9. Zhang, X.; Wu, S.; Wu, B.; Yang, Q.; Chen, A.; Li, Y.; Zhang, Y.; Pan, T.; Zhang, H.; He, X. SARS-CoV-2 Omicron Strain Exhibits Potent Capabilities for Immune Evasion and Viral Entrance. *Sig Transduct Target Ther* **2021**, *6*, 1–3, doi:10.1038/s41392-021-00852-5.
10. Kim, S.; Liu, Y.; Lei, Z.; Dicker, J.; Cao, Y.; Zhang, X.F.; Im, W. Differential Interactions between Human ACE2 and Spike RBD of SARS-CoV-2 Variants of Concern. *J Chem Theory Comput* **2021**, *17*, 7972–7979, doi:10.1021/acs.jctc.1c00965.
11. Kim, S.; Liu, Y.; Ziarnik, M.; Seo, S.; Cao, Y.; Zhang, X.F.; Im, W. Binding of Human ACE2 and RBD of Omicron Enhanced by Unique Interaction Patterns among SARS-CoV-2 Variants of Concern. *J Comput Chem* **2023**, *44*, 594–601, doi:10.1002/jcc.27025.
12. Han, P.; Li, L.; Liu, S.; Wang, Q.; Zhang, D.; Xu, Z.; Han, P.; Li, X.; Peng, Q.; Su, C.; et al. Receptor Binding and Complex Structures of Human ACE2 to Spike RBD from Omicron and Delta SARS-CoV-2. *Cell* **2022**, *185*, 630-640.e10, doi:10.1016/j.cell.2022.01.001.
13. Meng, B.; Abdullahi, A.; Ferreira, I.A.T.M.; Goonawardane, N.; Saito, A.; Kimura, I.; Yamasoba, D.; Gerber, P.P.; Fatihi, S.; Rathore, S.; et al. Altered TMPRSS2 Usage by SARS-CoV-2 Omicron Impacts Infectivity and Fusogenicity. *Nature* **2022**, *603*, 706–714, doi:10.1038/s41586-022-04474-x.
14. Cameroni, E.; Bowen, J.E.; Rosen, L.E.; Saliba, C.; Zepeda, S.K.; Culap, K.; Pinto, D.; VanBlargan, L.A.; De Marco, A.; di Iulio, J.; et al. Broadly Neutralizing Antibodies Overcome SARS-CoV-2 Omicron Antigenic Shift. *Nature* **2022**, *602*, 664–670, doi:10.1038/s41586-021-04386-2.
15. Mannar, D.; Saville, J.W.; Zhu, X.; Srivastava, S.S.; Berezuk, A.M.; Tuttle, K.S.; Marquez, A.C.; Sekirov, I.; Subramaniam, S. SARS-CoV-2 Omicron Variant: Antibody Evasion and Cryo-EM Structure of Spike Protein-ACE2 Complex. *Science* **2022**, eabn7760, doi:10.1126/science.abn7760.
16. Lan, J.; Ge, J.; Yu, J.; Shan, S.; Zhou, H.; Fan, S.; Zhang, Q.; Shi, X.; Wang, Q.; Zhang, L.; et al. Structure of the SARS-CoV-2 Spike Receptor-Binding Domain Bound to the ACE2 Receptor. *Nature* **2020**, *581*, 215–220, doi:10.1038/s41586-020-2180-5.
